# Alpha peak frequency affects visual performance beyond temporal resolution

**DOI:** 10.1162/imag_a_00107

**Published:** 2024-03-11

**Authors:** Maëlan Q. Menétrey, Maya Roinishvili, Eka Chkonia, Michael H. Herzog, David Pascucci

**Affiliations:** Laboratory of Psychophysics, Brain Mind Institute, École Polytechnique Fédérale de Lausanne (EPFL), Lausanne, Switzerland; Laboratory of Vision Physiology, Ivane Beritashvili Centre of Experimental Biomedicine, Tbilisi, Georgia; Institute of Cognitive Neurosciences, Free University of Tbilisi, Tbilisi, Georgia; Department of Psychiatry, Tbilisi State Medical University (TSMU), Tbilisi, Georgia

**Keywords:** alpha oscillations, individual peak frequency, visual processing, temporal resolution

## Abstract

Recent work suggests that the individual alpha peak frequency (IAPF) reflects the temporal resolution of visual processing: individuals with higher IAPF can segregate stimuli at shorter intervals compared to those with lower IAPF. However, this evidence mainly comes from studies focusing on short intervals, with stimulus onset asynchronies (SOA) rarely extending beyond a single alpha cycle (e.g., ~100 ms). Here, we investigated the relationship between IAPF and performance in visual backward masking (VBM), which allowed us to test the effects of IAPF for longer SOAs than an alpha cycle. A group of healthy controls (N = 79) and schizophrenia patients (N = 121), who generally exhibit lower IAPF, were tested in conditions with a Vernier shown alone, a Vernier followed by a mask at two SOAs (30 and 150 ms), or only a mask. Our results show that IAPF can predict VBM performance in all conditions with a Vernier. Furthermore, in both the control and schizophrenia groups, individuals with higher IAPF showed reduced masking effects, even when the SOA of 150 ms exceeded the alpha cycle. These findings challenge the notion that IAPF is exclusively related to temporal resolution and visual processing within a single alpha cycle. We discuss alternative mechanisms by which IAPF determines visual performance.

## 1 Introduction

Recent research suggests a link between occipital alpha rhythms (8-13 Hz;Adrian & Matthews, 1934;Berger, 1929) and the temporal resolution of visual perception (Ronconi et al., 2018;Samaha & Postle, 2015;VanRullen, 2016). Individuals with a higher individual alpha peak frequency (IAPF), which represents the frequency with the highest alpha power, tend to perform better at segregating two stimuli with short stimulus-onset asynchronies (SOA) compared to individuals with a lower IAPF (for a meta-analysis, seeSamaha & Romei, 2023).

It is believed that stimuli falling within the same alpha cycle are integrated and perceived as a single event (Samaha & Postle, 2015;VanRullen & Koch, 2003). Thus, a higher IAPF, characterized by shorter alpha cycles, would result in narrower integration windows and therefore better temporal resolution. According to this view, the effects of IAPF should be only observable when two stimuli are presented with Short SOAs so that the two stimuli can fall within a single alpha cycle (Wutz & Melcher, 2014). Consequently, many studies used paradigms like two-flash fusion, where two brief flashes are separated by SOAs that rarely exceed the typical alpha cycle (e.g., 100 ms;Battaglini et al., 2020;Deodato & Melcher, 2023;Gray & Emmanouil, 2020;Ronconi & Melcher, 2017;Samaha & Postle, 2015; but seeDrewes et al., 2022).

**Fig. 1.**
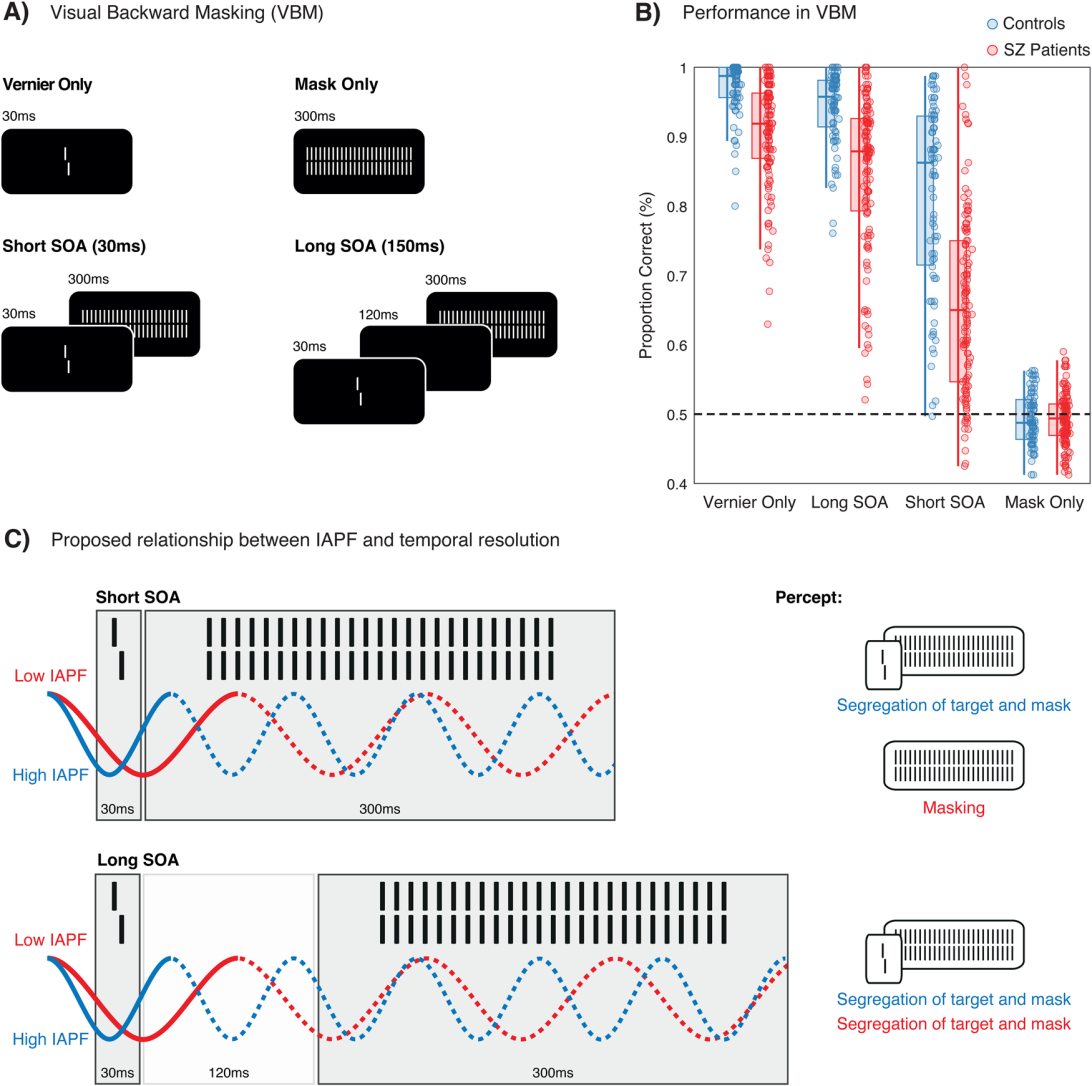
Participants reported whether the lower line of the Vernier was offset either to the left or right (as shown here). (A) In the Vernier Only condition, the Vernier is presented alone for 30 ms. In the Mask Only condition, only the mask is presented for 300 ms. In the Short and Long SOA conditions, the Vernier is followed by a mask with an SOA of either 30 or 150 ms. (B) Performance (proportion of correct responses) for all conditions. In comparison to controls (blue box plots and dots), SZ patients (red box plots and dots) exhibit deteriorated performance in all conditions with a Vernier, especially in the Short SOA condition. (C) According to the proposed relationship between IAPF and temporal resolution, masking effects should be evident only at the Short SOA of 30 ms. In this condition, performance—that is, the ability to segregate the target from the mask—should depend on the IAPF: higher IAPF (in blue) facilitates the segregation of the target from the mask because the two are more likely to fall in different alpha cycles; conversely, a lower IAPF (in red) does not allow to segregate the two, therefore leading to stronger masking effects. In the Long SOA condition, where the SOA extends beyond the alpha cycle, no masking effects are expected, and therefore no relationship between performance and IAPF.

Here, we investigated the relationship between IAPF, estimated from resting-state electroencephalography recordings (EEG), and performance in a visual backward masking paradigm (VBM; seeFig. 1A-B). In VBM, a target is followed by a mask, which impedes performance on the target depending on the SOA between the two (Breitmeyer & Ogmen, 2000). We reanalyzed a dataset (da Cruz et al., 2020;Garobbio et al., 2021;Gordillo et al., 2023) where participants performed four, randomly interleaved conditions: one with a Vernier target presented alone, two with the Vernier followed by a mask at two SOAs (Short SOA: 30 ms and Long SOA: 150 ms), and one with only the mask (Fig. 1A). In all conditions, participants had to report the perceived offset of the Vernier (left vs. right), even when only a mask was presented.

According to existing theories (Ronconi et al., 2018;Samaha & Postle, 2015;VanRullen, 2016), masking effects, resulting from the integration of the target and the mask, should occur within the window of an alpha cycle, with the length of this integration window depending on the IAPF. In the Short SOA trials, both the Vernier and the mask fall within a single alpha cycle, and thus, masking effects should correlate with IAPF (Fig. 1C). In the Long SOA condition, the Vernier and mask do not fall within the same alpha cycle, and therefore, there should be no masking effects (Fig. 1C).

We tested these predictions in a group of healthy controls (N = 79) and a group of schizophrenia (SZ) patients (N = 121). We included SZ patients since it is well known that they exhibit lower IAPF (Harris et al., 2006;Karson et al., 1988;Murphy & Öngür, 2019;Ramsay et al., 2021;Trajkovic et al., 2021;Yeum & Kang, 2018) and, in VBM, they require longer SOAs to achieve performance levels comparable to controls (Chkonia et al., 2010). Furthermore, SZ patients exhibit impaired performance in both the Short and Long SOA conditions (Favrod et al., 2018;Plomp et al., 2013; seeFig. 1B), indicating masking effects that extend beyond one alpha cycle.

To preface our results, we found that IAPF correlates with performance across all conditions with a Vernier. Specifically, we show that 1) masking effects occur also for SOAs longer than the alpha cycle in both groups and 2) IAPF predicts masking effects, even when the SOA is long. Furthermore, our results indicate that a higher IAPF corresponds to weaker masking effects in both healthy controls and schizophrenia patients. This suggests a relationship between IAPF and visual performance beyond the temporal resolution of a single alpha cycle. We discuss several alternative mechanisms that can explain our results.

## 2 Methods

### 2.1 Participants

We reanalyzed data from a group of SZ patients and healthy controls recruited from the Tbilisi Mental Health Hospital and the general population in Tbilisi (121 SZ patients, average age = 35.8 ± 9.2; 22 females; 79 healthy controls, average age = 34.9 ± 7.9; 42 females, seeTable 1). All procedures were approved by the local ethics committee and complied with the Declaration of Helsinki. Before the experiment, participants signed informed consent and were informed that they could stop the experiment at any time.

**Table 1.** Group average statistics (±SD) of SZ patients and controls.

	SZ patients	Controls
Gender (F/M)	22/99	42/37
Age (years)	35.8 ± 9.2	34.9 ± 7.9
Education (years)	13.3 ± 2.6	15.2 ± 2.9
Handedness (L/R)	115/6	73/6
Visual acuity	1.42 ± 0.4	1.6 ± 0.4
Illness duration (years)	10.8 ± 8.7	
SANS	10.1 ± 5.2	
SAPS	8.6 ± 3.2	
CPZ equivalent	561.1 ± 389.4	

Note: Visual acuity = Binocular visual acuity measured by the Freiburg Visual Acuity Test; SANS = Scale for the Assessment of Negative Symptoms; SAPS = Scale for the Assessment of Positive Symptoms; CPZ = Chlorpromazine equivalents (calculated over the 106 SZ patients receiving neuroleptic medication).

The data were obtained from previous studies conducted with the same participants, involving EEG recordings during a VBM task (da Cruz et al., 2020;Garobbio et al., 2021) and a 5-min resting-state period (Gordillo et al., 2023). All the data were collected on the same day, with the resting-state EEG recorded before the VBM task. All participants had normal binocular visual acuity of at least 0.8, as measured by the Freiburg Visual Acuity Test (Bach, 1996).

SZ patients were included based on prior diagnosis and provided that they had sufficiently recovered from an acute episode. Controls were recruited from the general population, aiming to match SZ patients as closely as possible in terms of age, gender, education, and visual acuity (seeTable 1).

### 2.2 Stimuli and apparatus

Stimuli were displayed on a Siemens Fujitsu P796-1 monitor (31.0 cm (H) × 23.3 cm (V)) with a refresh rate of 100 Hz. The screen resolution was 1024 × 768 pixels. Participants sat in a dimly illuminated room, 3.5 m away from the monitor. The study used a Vernier stimulus which consisted of two vertical line segments of 10′ (arcminutes) length separated by a vertical gap of 1′. The lower line was slightly offset randomly either to the left or to the right compared to the upper one, with a fixed horizontal gap of 1.2′ between them.

### 2.3 Visual backward masking task

To ensure comparable visual stimulation for all participants, the same Vernier duration, and Vernier-mask SOAs were used for all participants. The duration of the Vernier stimulus was fixed to 30 ms based on previous studies (Chkonia et al., 2010,^2012^;Herzog et al., 2004). The 25-element mask, presented for 300 ms, followed the Vernier with two possible SOAs: 30 ms, corresponding to the mean performance level of controls and 150 ms, corresponding to the mean performance level of SZ patients (Chkonia et al., 2010;Favrod et al., 2018).

As in previous works (da Cruz et al., 2020;Favrod et al., 2018;Garobbio et al., 2021;Plomp et al., 2013), four different experimental conditions were tested: Vernier Only (i.e., only the Vernier is presented), Long SOA (i.e., the Vernier is followed by a mask, after an interstimulus interval of 120 ms), Short SOA (i.e., the Vernier is directly followed by a mask), and Mask Only (i.e., only the mask is presented; seeFig. 1A). These conditions were tested in a pseudo-random order (20 trials per condition across 8 blocks), resulting in a total of 160 trials per condition. The inter-trial pause varied randomly between 1000 and 1500 ms. Vernier offset direction (left/right) was chosen pseudorandomly in each trial containing a Vernier, such that half the trials had a left/right offset. Participants reported the perceived offset direction of the lower bar compared to the upper bar of the Vernier stimuli by hand-held button presses (left vs. right). When uncertain, participants guessed the direction. Accuracy was emphasized over speed (Fig. 1B). In the Mask Only condition, response accuracy was determined by comparing the left/right offset to a randomly chosen notional offset, serving as a control condition.

### 2.4 EEG recordings and preprocessing

During resting-state recording, participants were instructed to keep their eyes closed and to relax while 5 min of recordings were collected in the total absence of visual stimulation. EEG was recorded using a BioSemi Active Two system with 64 Ag-AgCl sintered active electrodes distributed across the scalp according to the 10/20 layout system. The sampling rate was 2048 Hz. Offline data were pre-processed in MATLAB (R2012a, The MathWorks, Inc., Natick, MA) using an automatic preprocessing pipeline (seeda Cruz et al., 2018), including the following steps: downsampling to 256 Hz, high-pass filtering (1 Hz cutoff frequency), and removal of power line noise; robust re-referencing to the biweight estimate of the mean of all channels; detection, removal, and 3D spline interpolation of bad electrodes; detection and removal of bad epochs; detection and removal of eye movement-, muscular-, and bad channel-related artifacts based on independent component analysis (ICA); and detection, removal, and interpolation of bad channels in epochs. The clean EEG data were then re-referenced to the average reference. We removed the first and last 30 sec of the 5 min recording and defined artificial epochs of 4 sec. On average, 1.5 channels per EEG recording were interpolated. The proportion of rejected epochs was about 6% per participant. Similar preprocessing was applied to the EEG data recorded during the VBM task. We extracted epochs from -1000 ms to 300 ms after stimulus onset. On average, 6 channels per EEG recording were interpolated. The proportion of rejected trials was about 4% per participant. In total, two SZ patients and four controls were excluded after preprocessing due to excessive muscular artifacts or bad electrodes.

### 2.5 Estimation of IAPF

**Fig. 2.**
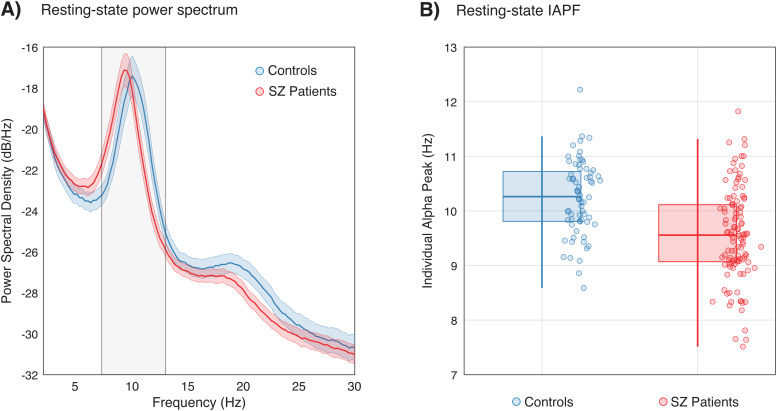
(A) Resting-state EEG power spectral density measured in controls (blue) and SZ patients (red). The gray area corresponds to the range of frequencies used for estimating the IAPF. Shaded areas are 95% CI. (B) Distribution of IAPF, measured from all electrodes, across the two groups. IAPF in controls (M = 10.25 Hz; blue box plots and dots) and SZ patients (M = 9.56 Hz; red box plots and dots) differ significantly (*p*< .001, Cohen's*d*= 0.88).

The IAPF was estimated from the power spectral density (PSD) of resting-state EEG recordings obtained via fast Fourier transform (*fft()*, MATLAB R2022b), computed across all electrodes. For each participant, IAPF was identified following a data-driven approach (Ouyang et al., 2020): first, we averaged the PSD across channels and resting-state segments; then, we estimated and removed the 1/f trend of the PSD via log-linear regression (Becker et al., 2018); and lastly, we fitted a Gaussian function to estimate the peak PSD in the alpha range (Fig. 2A-B). The alpha range of interest was restricted to 7.25-13 Hz (Ramsay et al., 2021). Participants with estimated peaks below or above this range were excluded (six SZ patients and seven controls). In total, 181 participants (113 SZ patients and 68 controls) were included in the analyses.

To evaluate the generalizability of the IAPF results across the resting-state session and the VBM task, we also used the EEG recordings during the VBM task to estimate the IAPF in the pre-stimulus interval of each trial (-1000 ms to 0 ms; seeSupplementary Material). In addition, we investigated time-varying estimates of instantaneous alpha frequency using the frequency-sliding window approach (Cohen, 2014;Menétrey et al., 2023; seeSupplementary Material).

### 2.6 Analyses

We first compared performance (proportion correct) between conditions and groups by means of paired and two-sample t-tests to examine the impact of masking on Vernier discrimination (seeResultssection andFig. 1B). In addition, we conducted Spearman correlations in each group to assess the relationship between performances in the different conditions (seeResultssection).

**Fig. 3.**
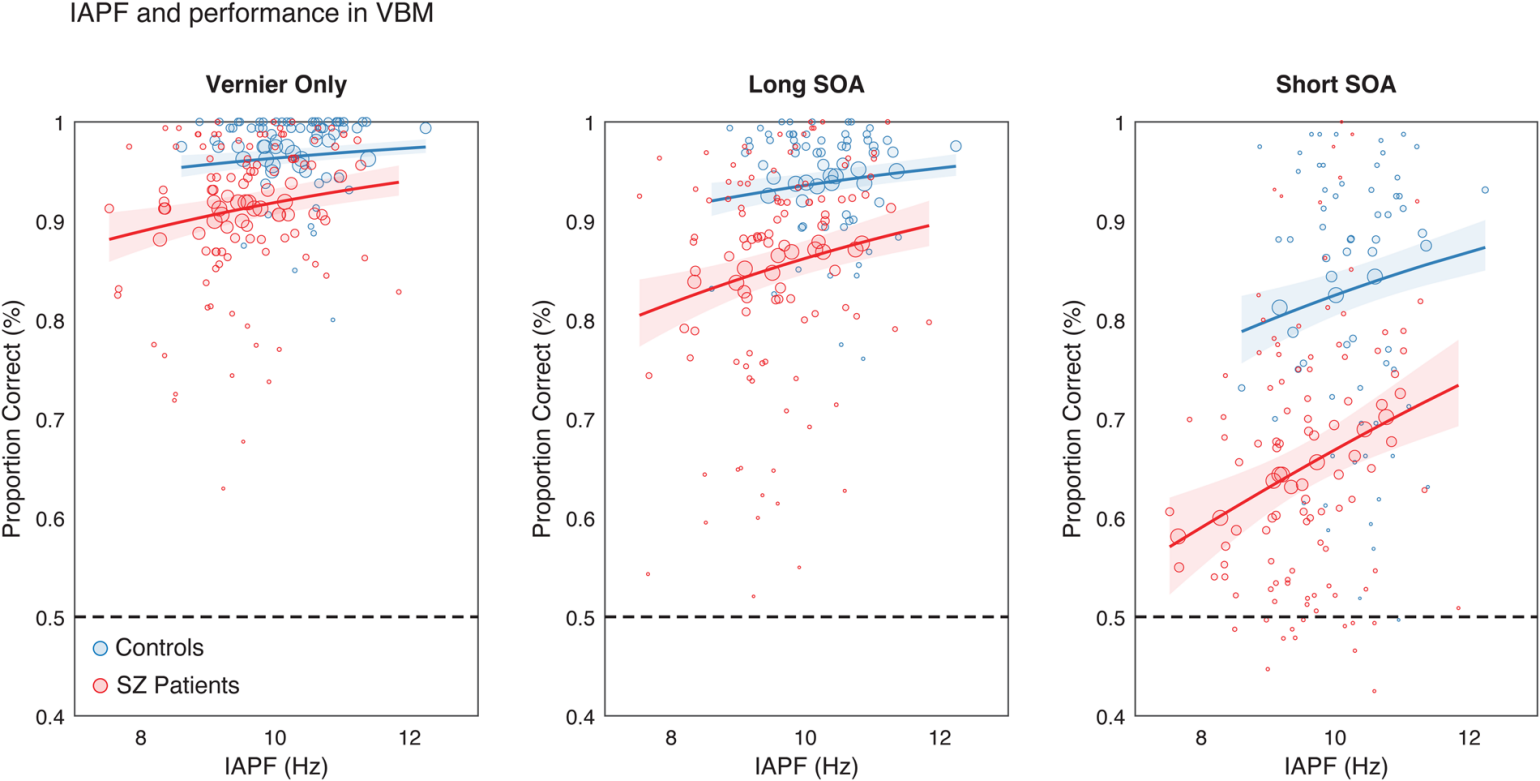
Generalized linear model (GLM) evaluating VBM performance as a function of IAPF, Group, and Condition (predictors selected by a stepwise procedure). IAPF significantly predicts performance (proportion correct) in all VBM conditions presenting a Vernier (Vernier Only, Long SOA, and Short SOA). The model's prediction is shown for each condition separately. Dots represent individual data points for the SZ (red) and control (blue) groups, varying inversely in size with the distance from the prediction curve. Lines and shaded areas are the predictions and 95% CI from the GLM.

To investigate the relationship between IAPF and performance, we focused on all conditions with a Vernier target and ran a stepwise model selection within the framework of a generalized linear regression model for proportional data (GLM), using a binomial distribution for the response variable (proportion correct) and a logit link with a free dispersion parameter (implemented with the*stepwiseglm()*function in MATLAB R2022b). The full model included the resting-state IAPF, a categorical variable coding for the Group (SZ patients vs. controls), a categorical variable coding for the Condition (Vernier Only vs. Long SOA vs. Short SOA), and all the possible interactions as the main predictors. The stepwise selection approach was employed with the aim to eliminate redundant or non-informative variables from the model (Table 2; seeStatistical analysessection). The predictions of performance derived from the GLM were plotted separately for each group in the three different conditions (Fig. 3).

**Table 2.** Results of the GLM evaluating VBM performance.

	Performance in VBM			
Predictors	Est.	SE	*t*	*p*
(Intercept)	1.57	0.45	3.45	**<.001**
IAPF	0.17	0.04	3.87	**<.001**
Group	-0.84	0.08	-9.75	**<.001**
Condition __LongSOA_	-0.58	0.1	-5.54	**<.001**
Condition __ShortSOA_	-1.72	0.09	-17.91	**<.001**

Note: Est. = Estimates (β), SE = Standard Error,*t*=*t*-statistic,*p*=*p*-value.

Significant effects are highlighted in bold.

**Fig. 4.**
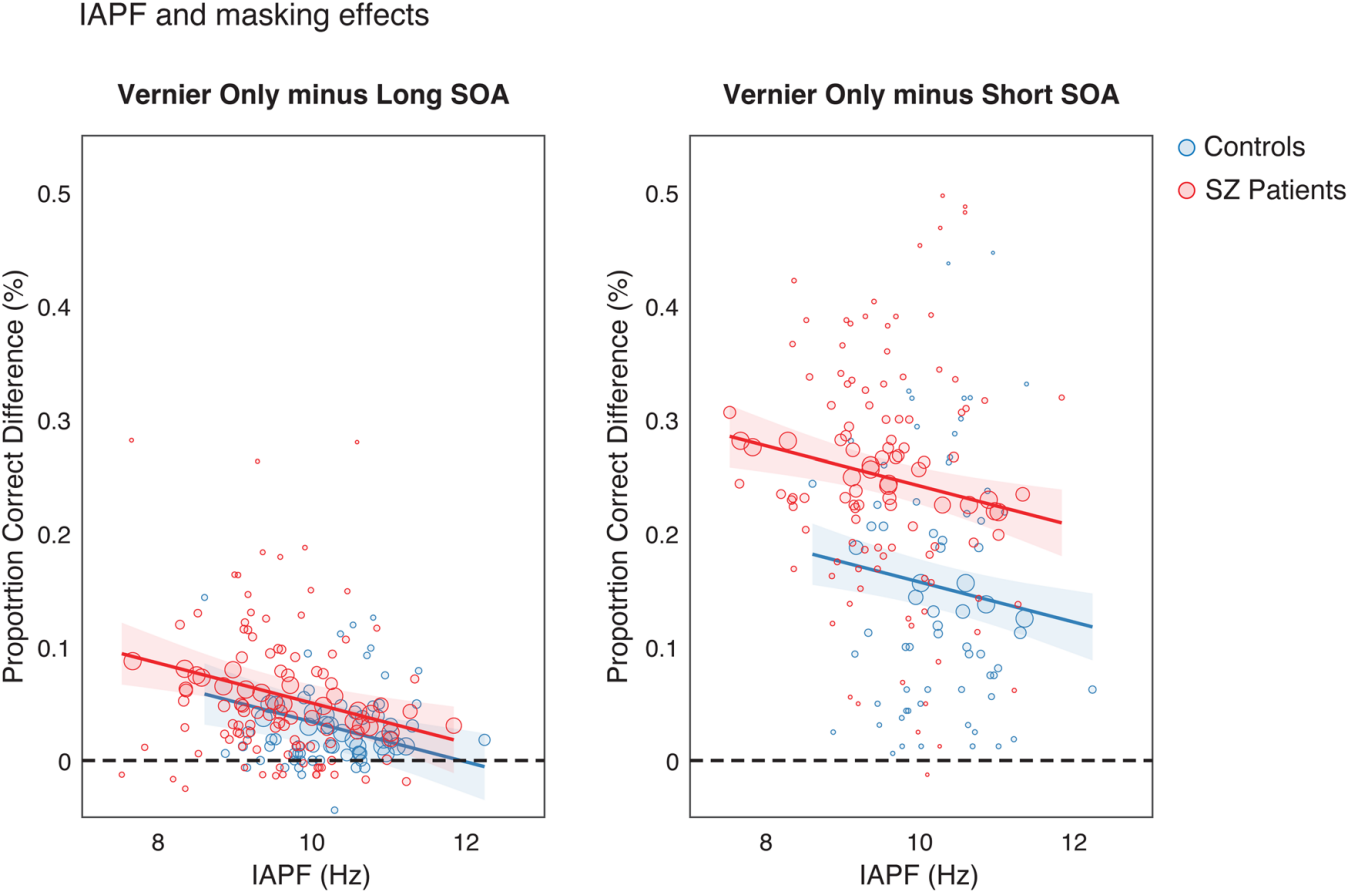
Linear model (LM) evaluating masking effects as a function of IAPF, Group, SOA, and Group*SOA interaction (predictors selected by a stepwise procedure). IAPF, SOA, and the interaction SOA*Group predict the intensity of masking effects, computed as the difference in performance (proportion correct) between the Vernier Only condition and the Long SOA or Short SOA conditions. The model's prediction is shown for each condition separately. Dots represent individual data points for the SZ (red) and control (blue) groups, varying inversely in size with the prediction curve. Lines and shaded areas are the predictions and 95% CI of the predictions from the LM.

We then tested whether IAPF is specifically related to masking effects by computing the difference between the Vernier Only condition (used as the baseline performance) and the two conditions with a mask (Long SOA and Short SOA). Differences in relative performance (masking effects) were modeled using a linear regression model (LM) with IAPF, Group, and SOA (Short vs. Long) as main predictors, along with their interactions, in a stepwise procedure similar to the one described above (Table 3;*stepwiselm*() function in MATLAB R2022b; seeStatistical analysessection). The LM predictions of masking effects were graphically illustrated separately for each group (Fig. 4).

**Table 3.** Results of the LM evaluating masking effects (i.e., relative performance between Vernier Only and Short/Long SOA conditions).

	Masking effects			
Predictors	Est.	SE	*t*	*p*
(Intercept)	0.2	0.05	3.61	**<.001**
IAPF	-0.01	0.005	-3.15	**.001**
Group	0.01	0.01	1.24	.21
SOA	0.12	0.01	8.65	**<.001**
Group:SOA	0.06	0.01	3.77	**<.001**

Note: Est. = Estimates (β), SE = Standard Error,*t*=*t*-statistic,*p*=*p*-value.

Significant effects are highlighted in bold.

### 2.7 Statistical analyses

All statistical comparisons and correlations related to behavioral performance were subjected to correction for multiple comparisons using the Bonferroni-Holm method (*bonf_holm*() function in MATLAB R2022b). Significance was determined if the*p*-value remained below 0.05 after correction. As estimates of effect size, we reported the Cohen’s*d*.

In the GLM and LM analyses, we adopted a stepwise procedure with backward selection. In the GLM, we started with a full model including all predictors and interactions and iteratively removed the least contributive predictors. The selection was done based on the deviance criterion. Specifically, for a term to be retained, the*p*-value for an F-test assessing the change in deviance resulting from adding or removing the term needed to be less than or equal to 0.1 (default value in*stepwiseglm()*function, MATLAB R2022b). Similarly, the stepwise procedure in the LM followed a stepwise procedure based on the sum of squared errors. Predictors were kept if the*p*-value for an F-test evaluating the change in the sum of squared error resulting from adding or removing the term was lower or equal to 0.1 (default value in*stepwiselm()*function, MATLAB R2022b).

## 3 Results

In the VBM task, it has been shown previously that SZ patients exhibit lower performance compared to controls in all conditions with a Vernier target (Chkonia et al., 2012;da Cruz et al., 2020;Favrod et al., 2018;Plomp et al., 2013). Here, we additionally show that each group demonstrates clear masking effects (Fig. 1B). When compared to the Vernier Only condition, performance decreases significantly in both the Short SOA (controls: t(74) = 12.23,*p*< .001, Cohen’s*d*= 1.56; SZ patients: t(118) = 26.16,*p*< .001, Cohen’s*d*= 2.31) and the Long SOA condition (controls: t(74) = 6.82,*p*< .001, Cohen’s*d*= 0.6; SZ patients: t(118) = 10.12,*p*< .001, Cohen’s*d*= 0.61). While masking effects are more pronounced in SZ patients, especially in the Short SOA condition, these results indicate that masking can still manifest, in both groups, at longer SOA. In addition, we found strong correlations in performance across the three conditions with a Vernier for both the controls (*r*> 0.75,*p*< .001) and SZ patients (*r*> 0.65,*p*< .001). This implies that individuals performing well in one condition often perform similarly well in others.

To test whether differences in performance correspond to potential differences in IAPF, we first estimated the IAPF, over all channels, from the resting-state EEG recordings of each participant (seeMethodssection). In line with previous work (Murphy & Öngür, 2019;Ramsay et al., 2021), SZ patients showed lower IAPF (mean IAPF = 9.56) compared to healthy controls (mean IAPF = 10.25), with a large effect size (t(179) = 5.73,*p*< .001, Cohen's*d*= 0.88;Fig. 2A-B). A similar difference was found in the pre-stimulus IAPF, measured during the VBM task (t(143) = 3.94,*p*< .001, Cohen's*d*= 0.69;Supplementary Fig. 2).

Next, we predicted performance (proportion of correct responses) in the VBM via generalized linear models (GLM). A full stepwise GLM was run to predict performance in the three conditions that included a Vernier, as a function of resting-state IAPF, Group, Condition, and their interactions (seeMethodssection). The stepwise procedure retained as main predictors all the three variables, but not the interactions (Table 2). This final model revealed a significant slope for the effect of IAPF on performance (β = 0.17  ±  0.04, t(538) = 3.87,*p*< .001), in addition to significant effects of Group (β = -0.84  ± 0.08, t(538) = -9.75,*p*< .001) and Condition (Long SOA: β = -0.58  ± 0.1, t(538) = -5.54,*p*< .001; Short SOA: β = -1.72  ± 0.09, t(538) = -17.91,*p*< .001).

In addition to the effect of Group and Condition, which show that the task is generally more challenging for SZ patients and that masking conditions are more difficult for both groups, these results indicate that IAPF is related to performance across all three conditions presenting a Vernier (Fig. 3; seeSupplementary Table 1andSupplementary Fig. 1for a control analysis modeling performance in the mask Only condition). The absence of interaction effects also confirms that the influence of IAPF is not restricted to the Short SOA condition or a specific group. Moreover, a control analysis demonstrated that the effects of IAPF are not a mere consequence of differences in alpha power (seeSupplementary Table 2).

Importantly, we replicated these results using the IAPF from pre-stimulus activity during the VBM task (Supplementary Table 3andSupplementary Fig. 3), also accounting for potential variations in IAPF during the pre-stimulus time. In particular, these additional control analyses show that 1) pre-stimulus IAPF leads to highly comparable results to the resting-state IAPF when used in the GLM, 2) this holds true also when IAPF is computed from the instantaneous alpha frequency (Cohen, 2014; seeSupplementary Fig. 4for additional analysis also controlling for the relationship between instantaneous alpha frequency and correct/incorrect trials), and 3) the IAPF at rest strongly correlates with the pre-stimulus IAPF (*r*= 0.8;*p *< .001).

Together, these additional results support the stability of IAPF across rest and task conditions, also ruling out the potential confound of pre-stimulus fluctuations.

The GLM results presented so far indicate that a higher IAPF facilitates Vernier offset discrimination, independently of the presence of a mask. While these findings suggest a general performance effect, it remains plausible, based on previous research associating IAPF with temporal integration in the alpha cycle, that IAPF may also predict masking effects, particularly at short SOA. To explore this possibility, we employed a stepwise linear regression model (LM, seeMethodssection). The model aimed to predict performance differences (i.e., relative performance) between the Vernier Only condition and both Long and Short SOA conditions, incorporating IAPF, Group, SOA, and their interactions as predictors. If IAPF influences masking effects within the alpha cycle, we hypothesized that higher IAPF would mitigate the detrimental impact of the mask on performance compared to the Vernier Only condition, with a more pronounced effect at Short SOAs, resulting in an interaction SOA*IAPF.

The stepwise linear regression revealed IAPF (β = -0.01 ± 0.005, t(357) = -3.15,*p*= .001), Group (β = 0.01 ± 0.01, t(357) = 1.24,*p*= .21), SOA (β = 0.12 ± 0.01, t(357) = 8.65,*p*< .001), and the Group*SOA interaction (β = 0.06 ± 0.01, t(357) = 3.77,*p*< .001) as variables in the preferred model. Notably, the SOA*IAPF interaction was excluded (Table 3), suggesting that IAPF influences masking effects consistently across both Long and Short SOA conditions (Fig. 4; seeSupplementary Fig. 5and related mediation analysis for a control of potential confounds due to performance correlation between Long and Short SOA). Furthermore, the lack of any interaction with IAPF suggests that IAPF alone does not fully account for performance differences between groups, nor for the more pronounced masking effects at short SOA in patients.

## 4 Discussion

We investigated the relationship between resting-state IAPF and performance in a VBM paradigm with healthy controls and SZ patients (N = 200;da Cruz et al., 2020;Garobbio et al., 2021;Gordillo et al., 2023), two populations known to exhibit different IAPF on average (Ramsay et al., 2021). Prior research suggests that IAPF is linked to the temporal resolution of visual processing, defined by the duration of an alpha cycle (Drewes et al., 2022;Samaha & Postle, 2015;VanRullen, 2016). For this reason, most studies have used paradigms with SOAs shorter than the interval of an alpha cycle. Here, we show that IAPF predicts performance and masking effects even when the SOA between two stimuli exceeds a single alpha cycle.

Our results are important for two reasons. First, according to theories linking alpha cycles to windows of perceptual integration (Ronconi et al., 2018;Samaha & Romei, 2023;Wutz et al., 2018), the effects of IAPF should be only found within the temporal window of an alpha cycle. However, some studies have reported null findings (Buergers & Noppeney, 2022;Grabot et al., 2017;London et al., 2022;Ronconi et al., 2022; but seeSamaha & Romei, 2023for a meta-analysis) or raised some criticisms (Schoffelen et al., 2024), and others have provided unclear neural correlates (Hülsdünker & Mierau, 2021;Morrow et al., 2023). Crucially, prior studies did not explore SOAs longer than the alpha cycle, while our results, which are not based on null findings, indicate that IAPF is related to aspects of visual processing extending beyond the short window of an alpha cycle: IAPF can predict the ability to segregate a target from the following mask even at longer SOAs.

Second, several studies have consistently shown that both masking effects and lower IAPF are reliable characteristics of schizophrenia (e.g.,Chkonia et al., 2010;Ramsay et al., 2021), suggesting a common underlying dysfunction specific to the disease, characterized by longer windows of temporal integration (Chen et al., 2014;Keane et al., 2016;Parsons et al., 2013;Sponheim et al., 2023). However, our study challenges this idea by revealing that IAPF is not tied to specific windows of temporal integration. Instead, it affects visual performance regardless of the presence of the mask and this pattern held true for both healthy controls and SZ patients, indicating a general characteristic independent of the population. Furthermore, our analyses showed that SZ patients consistently performed worse than controls, even with a comparable IAPF, as evident inFigure 3(see alsoTable 2). This implies that the difference in IAPF alone cannot explain, entirely, poorer performance in schizophrenia, and additional factors are likely contributing.

Overall, IAPF appears associated with visual performance in all conditions with a Vernier, suggesting a role in enhancing target processing (Di Gregorio et al., 2022;Tarasi & Romei, 2023). However, our analyses also revealed a more specific influence of IAPF on masking effects in VBM, as individuals with higher IAPF showed reduced detrimental effects of backward masking. This was evident also in the Long SOA condition, indicating that the underlying mechanisms have to do with the interaction between the target and the mask over relatively extended time windows, and consequently cannot reflect a process confined within the alpha cycle (Samaha & Postle, 2015;Wutz & Melcher, 2014).

It remains plausible that paradigms involving backward masking and integration/segregation (e.g., two-flash fusion) may engage partially distinct mechanisms. For instance, masking could interfere with the ongoing processing of the target without leading to a perceptual integration of the target and mask. While there is evidence that backward masking involves an early integration of sensory signals related to the target and mask (Gale et al., 2023), it is also important to note that prior studies have reported stronger masking effects with SOA between 50 and 100 ms (Bacon-Macé et al., 2005;Breitmeyer & Ganz, 1976;Ro, 2019). Crucially, these findings were previously interpreted to support temporal integration within the alpha cycle (Samaha & Romei, 2023). Our results indicate that, whether masking effects reflect integration or interference, the association between IAPF and masking in VBM extends beyond the alpha cycle. In line with our findings, other studies have demonstrated that there is no consistent relationship between IAPF and the interstimulus interval at which an observer perceives two stimuli as separate percepts (e.g.,Buergers & Noppeney, 2022;London et al., 2022;Samaha & Postle, 2015;Shen et al., 2019). Such variability, likely due to differences in tasks and paradigms, supports the notion that there is no fixed window of temporal integration dictated by the IAPF. Thus, alternative explanations are required, beyond those focusing solely on temporal resolution and integration windows.

We propose several candidate mechanisms to explain our findings, all of which are linked to the speed of neural processing. Specifically, a higher IAPF has been associated with shorter propagation delays due to a greater density of white matter (Valdés-Hernández et al., 2010). Such stronger wiring may lead to more efficient neuronal communication and faster transmission of target information to higher-level processing stages (Lőrincz et al., 2009;Ociepka et al., 2022). Secondly, higher IAPF may indicate quicker recurrent processing, reducing the interference of feedforward signals elicited by the mask with the feedback from higher visual areas responding to the target (Breitmeyer & Ogmen, 2006;Ro, 2019). Lastly, higher IAPF could facilitate the rapid inhibition of irrelevant stimuli following a relevant target (Wöstmann et al., 2019), ultimately lessening the impact of the mask. All of these possibilities (faster relay of target information, speeded recurrent processing or rapid inhibition of irrelevant stimuli) draw support from the established role of alpha activity in coordinating communication (Lobier et al., 2018;Pagnotta et al., 2020,^2022^;Pascucci et al., 2018) and functional inhibition within brain networks (Jensen & Mazaheri, 2010;Klimesch et al., 2007;Peylo et al., 2021;Romei et al., 2010). Consistent with these findings, and with the lower visual performance in SZ patients, lower IAPF has also been related to reduced connectivity in individuals with high schizotypal personality traits (Trajkovic et al., 2021).

Thus, we propose that IAPF does not affect temporal integration/segregation, or more generally, it is not strictly related to the temporal resolution of perception and to processes constrained within a single alpha cycle. In visual masking, IAPF may influence the speed of processing and relay of target information, with a faster IAPF shielding the neural representation of the target from masking effects. Conversely, a lower IAPF might prolong the processing duration and persistence of sensory traces, facilitating interactions beyond the typical alpha cycle (Schoffelen et al., 2024; see alsoMenétrey et al., 2023). This suggests a more general relationship between IAPF and general visual abilities, aligning with various studies linking IAPF to diverse functions involved in task performance, not limited to temporal resolution (e.g.,Clark et al., 2004;Grandy et al., 2013;Klimesch et al., 1993,^1996^;Lebedev, 1994). Importantly, the proposed relationship between IAPF and processing speed provides a coherent explanation for the observed effects of IAPF in other paradigms like two-flash fusion (higher IAPF correlating with more reports of two flashes; e.g.,Samaha & Postle, 2015).

One might question the extent to which resting-state IAPF can account for ongoing neural mechanisms during behavioral tasks. Despite the smaller sample size, we replicated the same effect of IAPF on performance using the pre-stimulus IAPF measured during the task (seeSupplementary Material). Additionally, we showed a strong correlation between resting-state and task-based IAPF in participants with clear alpha peaks in both recordings (*r*= 0.8). This supports earlier findings regarding the consistency of IAPF measurements within individuals (Grandy et al., 2013;Popov et al., 2023) and the reliability of resting-state IAPF for investigating its relation with perceptual and cognitive functions (e.g.,Drewes et al., 2022;Ramsay et al., 2021;Tarasi & Romei, 2023).

It is important to note that we also controlled for variations of instantaneous alpha frequency within the pre-stimulus window, and whether these variations could be differentially related to correct and incorrect responses at the single trial level. However, we found no evident relationships, contrary to what was reported in other studies (Samaha & Postle, 2015;Wutz et al., 2018; seeSupplementary Material). This additional finding further casts doubt on the link between IAPF and temporal resolution, supporting a more general role in task performance (Klimesch, 1999).

In conclusion, we showed that higher IAPF is related to general visual performance, and higher IAPF mitigates the detrimental effect of backward masking, even at longer SOAs. These findings held true for both healthy controls and SZ patients. Therefore, our results challenge the idea that IAPF is exclusively related to the temporal resolution of visual perception and to processes constrained within a single alpha cycle. Instead, they suggest that the effects of IAPF on visual performance are a general characteristic, extending beyond restricted temporal windows.

## Supplementary Material

Supplementary Material

## Data Availability

The data supporting the findings of this study are available on the Open Science Framework (https://osf.io/ktem6/?view_only=).
