# Supplementary material for: Alpha peak frequency affects visual performance beyond temporal resolution
Source: Imaging Neurosci (Camb). 2024 Mar 11;2:imag-2-00107. doi: 10.1162/imag_a_00107 (PMC12247567; doi:10.1162/imag_a_00107)
Supplement: Supplementary Material [file imag_a_00107-supp.pdf]

Supplementary Material

Control analysis with the Mask Only condition

As performance was determined randomly in the Mask Only condition, we did not include this condition in our main GLM analysis (Table 2 and Figure 3). Nevertheless, to confirm the absence of any effect, we ran a stepwise GLM predicting performance in the Mask Only condition as a function of the IAPF, the Group variable, and their interaction. As expected, both the IAPF and Group variables were excluded from the preferred model, indicating no effect on performance (Supplementary Table 1 and Supplementary Figure 1).

Supplementary Table 1. Results of the GLM evaluating performance in Mask Only condition.

	Performance in Mask Only condition			
**Predictors**	**Est.**	**SE**	***t***	***p***
(Intercept)	-0.03	0.01	-2.9	**.004**

Note: Est. = Estimates (β), SE = Standard Error, *t* = *t*-statistic, *p* = *p*-value.

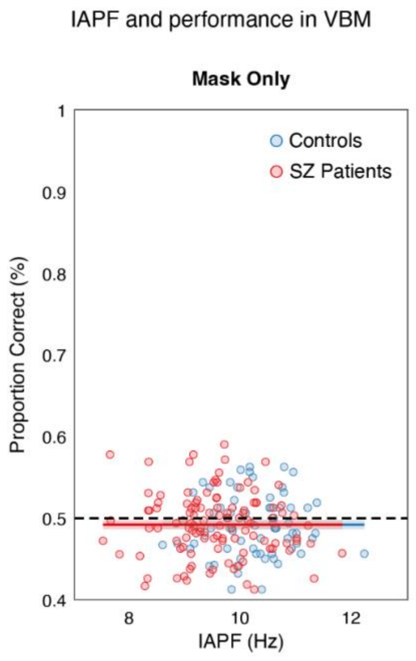

Supplementary Figure 1. Generalized linear model (GLM) evaluating performance in Mask Only condition. As performance in this condition is determined by comparing the response to a randomly chosen notional offset (left or right), IAPF and Group variables have no effect and are excluded from the final model. Dots represent individual data points for the SZ (red) and control (blue) groups. Lines and shaded areas are the predictions and 95% CI of the predictions from the GLM.

Control analysis for a confounding effect of alpha power

Alpha power is known to modulate sensory processing: stronger alpha power usually impairs the detection of brief stimuli (e.g., Brüers & VanRullen, 2018). Since alpha power and frequency may covary across individuals (Benwell et al., 2019), we verified that alpha power did not confound our main findings and that the effects reported were genuinely due to IAPF (Table 2 and Figure 3). To this aim, we rerun our main GLM after residualizing IAPF from any shared variance with the alpha power variable. Residualization was performed by taking the residuals of a linear model predicting IAPF with the power magnitude at the IAPF peak of each individual (see Supplementary Table 2). Residualization removes any shared variance between the two variables. This analysis showed that IAPF was significantly related to alpha power ($\beta = -0.05 \pm 0.02$, $t(181) = -3.08$, $p = .002$), thus representing a potential confound. However, the results of our main GLM remained consistent after using the IAPF residualized from the effects of power (IAPF_residuals), in place of the original IAPF variable (see Supplementary Table 2). Thus, even when controlling for alpha power, the influence of IAPF on performance remains significant ($\beta = 0.35 \pm 0.1$, $t(537) = 3.37$, $p < .001$).

Supplementary Table 2. Results of the LM evaluating VBM performance while controlling for power effects. Significant effects are highlighted in bold.

	IAPF			
**Predictors**	**Est.**	**SE**	***t***	***p***
(Intercept)	10.37	0.19	54.49	< **.001**
Alpha power	-0.05	0.02	-3.08	**.002**
	Performance in VBM			
(Intercept)	3.18	0.11	29.14	< **.001**
IAPF_residuals	0.35	0.1	3.37	< **.001**
Group	-0.79	0.08	-9.02	< **.001**
$Condition_{\_LongSOA}$	-0.59	0.1	-5.58	< **.001**
$Condition_{\_ShortSOA}$	-1.72	0.09	-18.02	< **.001**
Group * IAPF_residuals	-0.19	0.11	-1.65	.09

Note: Est. = Estimates (β), SE = Standard Error, *t* = *t*-statistic, *p* = *p*-value.

Analysis using pre-stimulus IAPF

Our main findings show that IAPF, measured during a resting-state period, affects performance in a VBM task. We aimed to generalize our results using the EEG recordings during the VBM task, and, specifically, estimating IAPF from the pre-stimulus interval of each trial (from -1000 ms to 0 ms). However, because data were noisier during the task, more than half of the subjects had to be excluded when considering all the electrodes, as they did not meet the criterion of a clear alpha peak. To increase the signal to noise, and following the procedure used in prior studies, we therefore estimated the IAPF at the two electrodes showing the strongest pre-stimulus alpha power in the left and right hemifield across participants (Supplementary Figure 2A-B). These corresponded to the electrodes A25 and B30. Using only these electrodes, a total of 145 participants was retained (95 SZ patients and 50 controls), and the group difference in IAPF remained largely significant ($t(143) = 3.94$, $p < .001$, Cohen's $d = 0.69$). While both groups exhibited slightly higher peaks compared to the resting-state IAPF (controls: 10.7 Hz instead of 10.25 Hz; SZ patients: 10 Hz instead of 9.56 Hz), we still observed a strong correlation between the two measures ($r = 0.8$; $p < .001$; Supplementary Figure 3A). Note that for this correlation analysis, we only included participants who exhibited an alpha peak in both resting-state and task recordings (N = 144; 94 SZ patients and 50 controls).

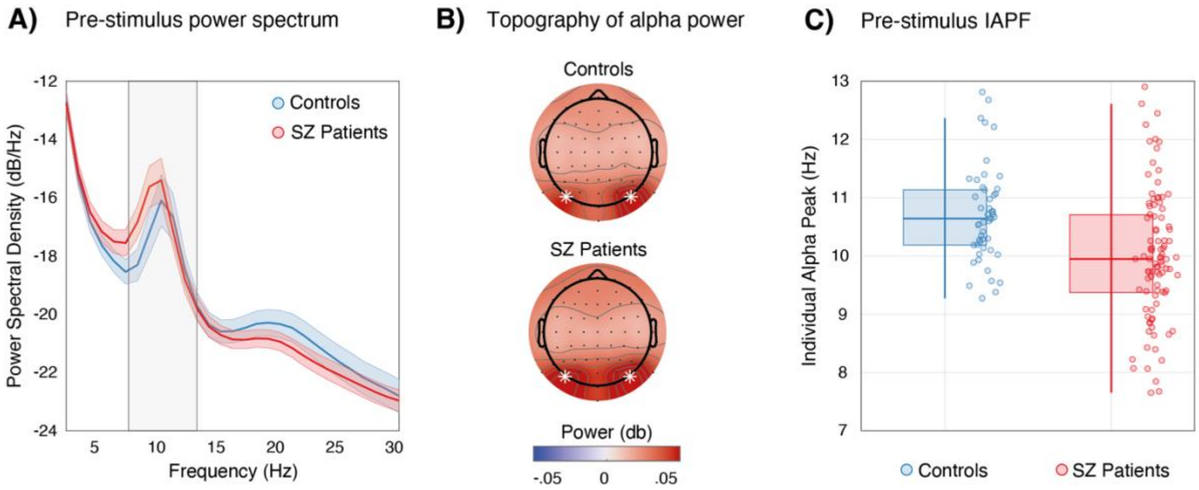

Supplementary Figure 2. A) Pre-stimulus EEG power spectral density measured in controls (blue) and SZ patients (red). The gray area corresponds to the range of frequencies used for estimating the IAPF. Shaded areas are 95% CI. B) The pre-stimulus alpha power topography in controls (upper plot) and SZ patients (lower plot). The white asterisks highlight the two electrodes exhibiting the strongest alpha power, which were the same in both groups (A25 and B30). C) Distribution of pre-stimulus IAPF across the two groups, measured from the occipital electrodes shown in B). IAPF in controls (M = 10.70 Hz; blue box plots and dots) and SZ patients (M = 10 Hz; red box plots and dots) differ significantly ($p < .001$, Cohen's $d = 0.69$).

Subsequently, we ran the stepwise GLM to predict performance across the three Vernier conditions, incorporating pre-stimulus IAPF, Group, Condition, and their interactions as predictors. The stepwise procedure kept only the three variables as main predictors (see Supplementary Table 3 and Supplementary Figure 3B). Consistent with our previous findings (Table 2 and Figure 3), these results revealed that pre-stimulus IAPF also has a consistent impact on visual performance, irrespective of the mask or the SOA between the target and the mask ($\beta = 0.09 \pm 0.03$, $t(430) = 2.55$, $p = .01$).

Supplementary Table 3. Results of the GLM evaluating VBM performance as a function of pre-stimulus IAPF. Significant effects are highlighted in bold.

	Performance in VBM			
**Predictors**	**Est.**	**SE**	***t***	***p***
(Intercept)	2.27	0.41	5.48	< **.001**
Pre-stimulus IAPF	0.09	0.03	2.55	**.01**
Group	-0.95	0.1	-9.63	< **.001**
$\text{Condition}_{\text{\_LongSOA}}$	-0.57	0.11	-4.89	< **.001**
$\text{Condition}_{\text{\_ShortSOA}}$	-1.66	0.1	-15.65	< **.001**

Note: Est. = Estimates (β), SE = Standard Error, *t* = *t*-statistic, *p* = *p*-value.

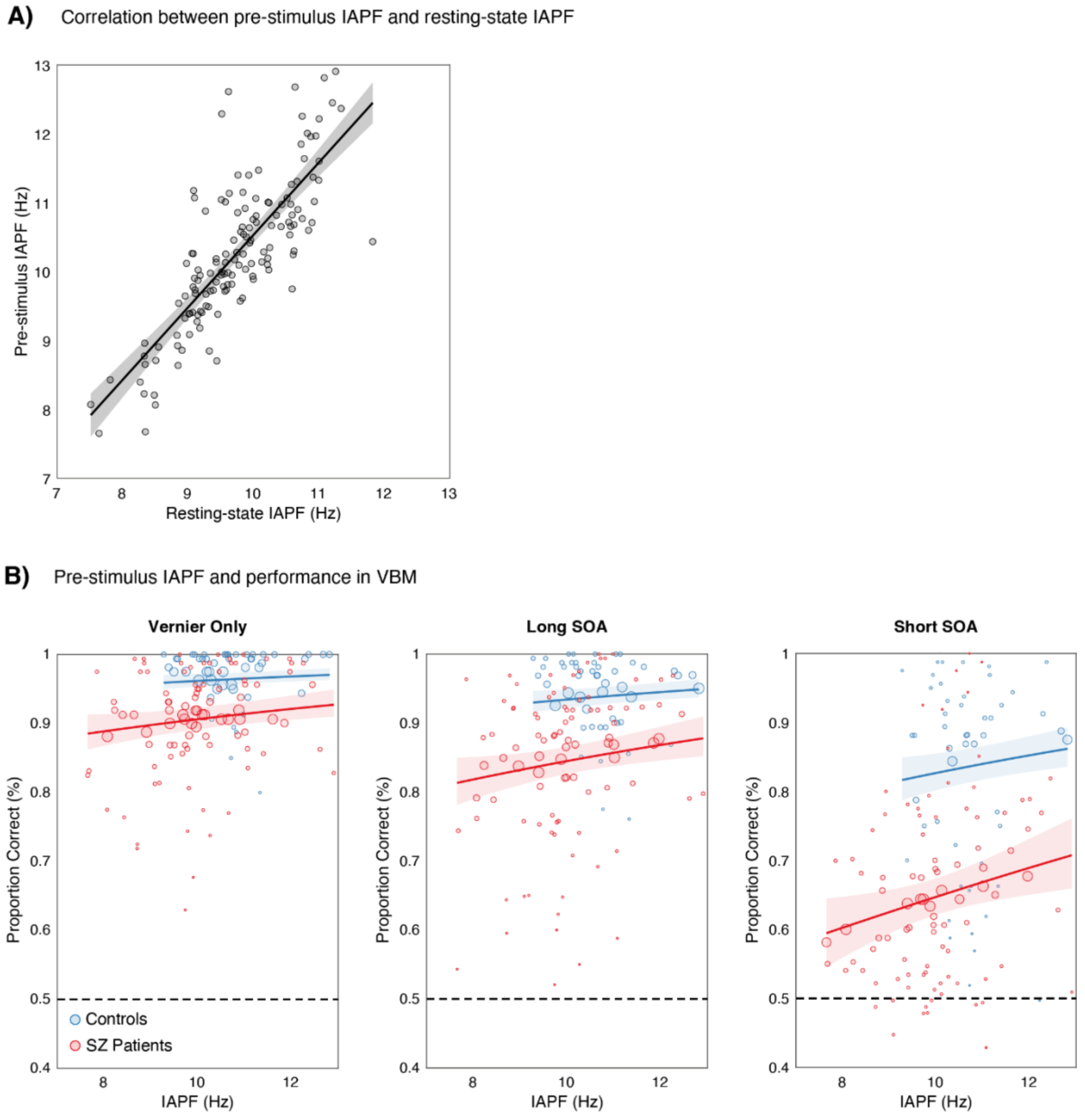

Supplementary Figure 3. A) Correlation between pre-stimulus IAPF and resting-state IAPF. The line and shaded areas are the predictions and 95% CI from a linear model. B) Generalized linear model (GLM) evaluating VBM performance as a function of pre-stimulus IAPF, Group, and Condition (predictors selected by a stepwise procedure). Pre-stimulus IAPF predicts performance (proportion correct) in all VBM conditions with a Vernier (Vernier Only, Long SOA and Short SOA). The model's prediction is shown for each condition separately. Dots represent individual data points for the SZ (red) and control (blue) groups, varying inversely in size with the prediction curve. Lines and shaded areas are the predictions and 95% CI of the predictions from the GLM.

Instantaneous frequency analysis in task EEG

We followed another method to analyze the alpha frequency in the EEG data recorded during the task. The frequency-sliding window approach (Cohen et al., 2014; Menétrey et al., 2023) allows a within-subjects analysis of the instantaneous alpha frequency. To compute it, we used the EEG signal from the two occipital electrodes shown in Supplementary Figure 2B. Each epoch underwent bandpass filtering within the alpha-band range (7.25 to 13 Hz), and the instantaneous phase angle was computed using the Hilbert transform. Next, the instantaneous alpha frequency was derived from the temporal rate at which the instantaneous phase angle changes (i.e., the temporal derivative of the instantaneous Hilbert phase, scaled by the sampling rate and 2*π). To mitigate noise-induced abrupt changes, 10 median filters were applied to the instantaneous frequency estimate (spanning 10 to 400 ms windows). Participants instantaneous alpha frequency was finally obtained by averaging the median frequency across all windows and over the two occipital electrodes (N = 194; 119 SZ patients and 75 controls).

Previous research suggested that subtle variations in pre-stimulus instantaneous frequency can predict perceptual integration in paradigms presenting two successive stimuli: higher alpha frequencies enhance the ability to segregate the two stimuli (Samaha & Postle, 2015; Wutz et al., 2018). To investigate this effect, we first examined whether pre-stimulus instantaneous frequency significantly differed between correct and incorrect reports in the Short SOA condition. This condition was specifically chosen since it is the only one allowing both the Vernier and mask to potentially fall within the same alpha cycle. Thus, if alpha cycles determine integration windows, any effect would likely manifest here. However, our analysis revealed no significant difference between correct and incorrect trials within the pre-stimulus period (from -1000 ms to 0 ms), even without correcting for multiple comparisons (Supplementary Figure 4A). This result provides additional evidence against a specific influence of alpha frequency on integration mechanisms.

Nevertheless, we found that pre-stimulus instantaneous frequency (averaged from -500 to 0 ms) displayed a relatively strong correlation with resting-state IAPF (r = 0.61; p < .001; Supplementary Figure 4B). Even when accounting for nearly the entire epochs (averaged from -900 to 200 ms to remove edge artifacts), this correlation remains stable (r = 0.61; p < .001). Note that for these correlation analyses, we only included participants who exhibited an alpha peak in their resting-state data (N = 181; 113 SZ patients and 68 controls).

Therefore, we ran a last stepwise GLM predicting performance across the three Vernier conditions using instantaneous frequency (averaged from -500 to 0 ms), Group, Condition, and

their interactions as main predictors. The stepwise procedure retained instantaneous frequency, Group, Condition, and one interaction (Supplementary Table 4 and Supplementary Figure 4C). Notably, this model revealed a significant effect of instantaneous frequency on performance ($\beta = 0.89 \pm 0.19$, $t(576) = 4.64$, $p < .001$). This indicates that, besides the significant effects of Group and Condition, the pre-stimulus instantaneous frequency during the task also predicts performance across all conditions, regardless of the mask or the SOA. In addition, the significant interaction between instantaneous frequency and Group ($\beta = -0.63 \pm 0.2$, $t(576) = -3.11$, $p = .002$) might suggest that this effect is stronger for the control group, but this was not confirmed with the resting-state or pre-stimulus IAPF.

Supplementary Table 4. Results of the GLM evaluating VBM performance as a function of instantaneous frequency. Significant effects are highlighted in bold.

	Performance in VBM			
**Predictors**	**Est.**	**SE**	***t***	***p***
(Intercept)	-5.71	1.92	-2.96	**.003**
Instantaneous frequency	0.89	0.19	4.64	**< .001**
Group	5.56	2.05	2.70	**.007**
$\text{Condition}_{\text{\_LongSOA}}$	-0.58	0.1	-5.84	**< .001**
$\text{Condition}_{\text{\_ShortSOA}}$	-1.72	0.09	-19.01	**< .001**
Inst. frequ. * Group	-0.63	0.2	-3.11	**.002**

Note: Est. = Estimates (β), SE = Standard Error, *t* = *t*-statistic, *p* = *p*-value.

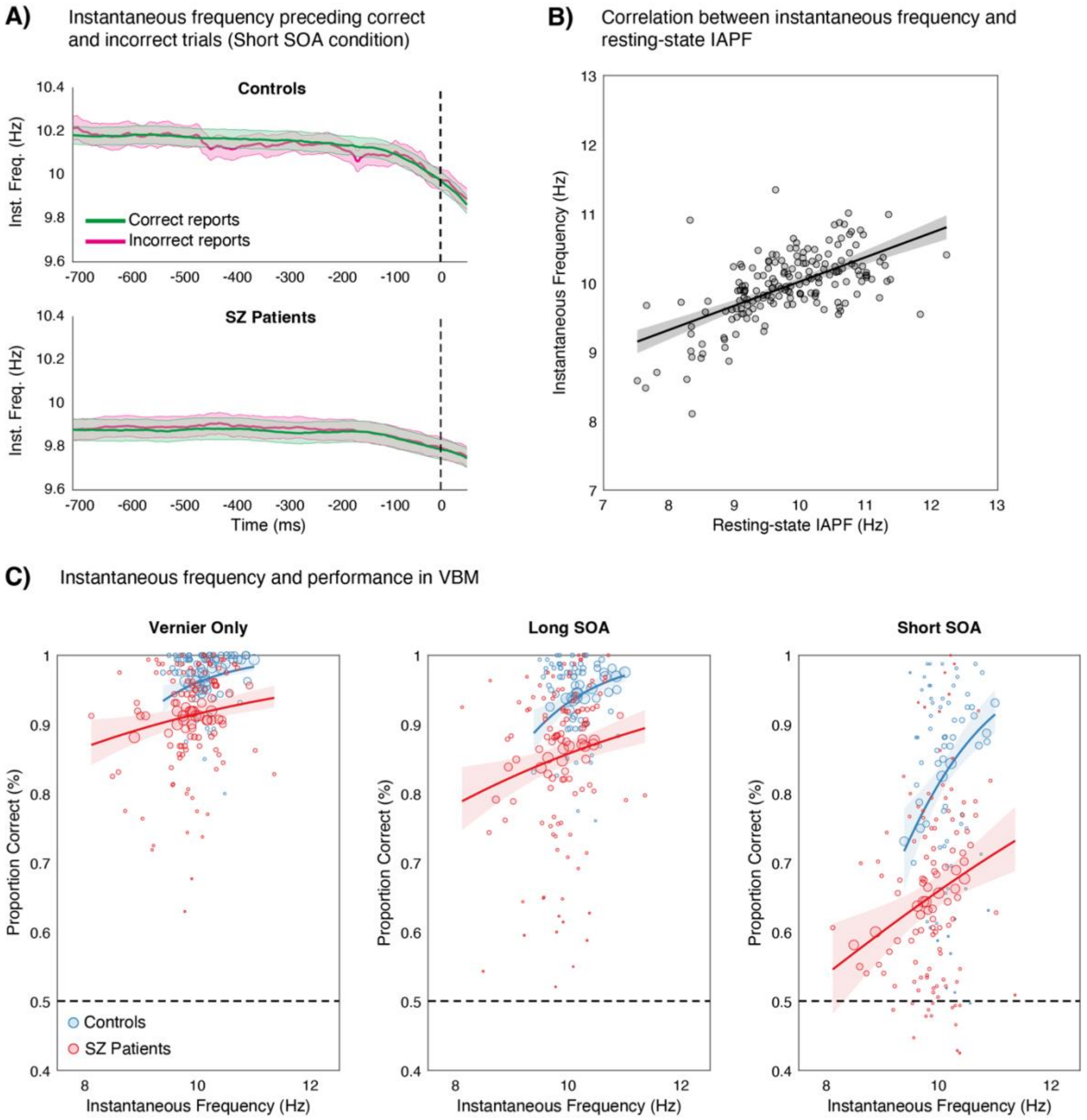

Supplementary Figure 4. A) Pre-stimulus instantaneous frequency in the Short SOA condition. Within-subjects comparisons, performed separately for each group (controls = upper plot; SZ patients = lower plot), showed no significant difference in alpha frequency preceding correct (green line) and incorrect (red line) reports ($p < .05$; uncorrected). Shaded areas represent SEM. B) Correlation between pre-stimulus instantaneous frequency and resting-state IAPF. The line and shaded areas are the predictions and 95% CI from a linear model. C) Generalized linear model (GLM) evaluating VBM performance as a function of instantaneous frequency, Group, Condition, and instantaneous frequency * Group interaction (predictors selected by a stepwise procedure). Instantaneous frequency significantly predicts performance (proportion correct) in all VBM conditions with a Vernier (Vernier Only, Long SOA and Short SOA). The model's prediction is shown for each condition separately. Dots represent individual data points for the SZ (red) and control (blue) groups, varying inversely in size with the prediction curve. Lines and shaded areas are the predictions and 95% CI of the predictions from the GLM.

Mediation analysis

Performance in the Vernier Only, Short SOA, and Long SOA conditions is highly correlated. Thus, one may wonder whether IAPF really affects the intensity of masking effects at both SOAs (shown with the LM results, see Table 3 & Figure 4), or whether the effects are just due to correlations between conditions. To rule out this potential confound, we performed a mediation analysis to determine whether the masking effects at Short SOA (i.e., defined by the relative performance difference between Vernier Only and Short SOA conditions) mediate the significant relationship found between resting-state IAPF and masking effects at Long SOA (i.e., defined by the relative performance difference between Vernier Only and Long SOA conditions; see Supplementary Figure 5). Using RStudio 2022.02.0 (R Core Team, 2022), a linear model was first applied to compute the effect of IAPF ($\beta = -0.01 \pm 0.005$, $t(178) = -2.26$, $p = .02$) and Group ($\beta = 0.02 \pm 0.008$, $t(178) = 2.36$, $p = .02$) on the masking effects in Long SOA. Second, a linear regression was performed to examine the relationship between IAPF ($\beta = -0.02 \pm 0.01$, $t(178) = -2.39$, $p = .02$) and masking effects in Short SOA, including the Group variable as additional predictor ($\beta = 0.08 \pm 0.01$, $t(178) = 4.6$, $p < .001$). Third, to control for the mediating role of Short SOA performance, we ran a final linear model to estimate the effect of IAPF ($\beta = -0.007 \pm 0.004$, $t(177) = -1.55$, $p = .12$), the mediating effect of performance in the Short SOA condition ($\beta = 0.16 \pm 0.03$, $t(177) = 4.5$, $p < .001$), and the Group effect ($\beta = 0.008 \pm 0.009$, $t(177) = 0.9$, $p = .37$) on masking effects in the Long SOA condition. Finally, the R package "medflex" was used to estimate direct and indirect effects (Steen et al., 2017). Despite observing a significant indirect effect, implying partial mediation of Short SOA on Long SOA performance ($\beta = -0.008 \pm 0.002$, $t(904) = -3.31$, $p = .001$), we also observed a significant direct effect of IAPF ($\beta = -0.01 \pm 0.004$, $t(904) = -2.6$, $p = .01$), thereby supporting a direct modulation of masking effects at Long SOA due to IAPF.

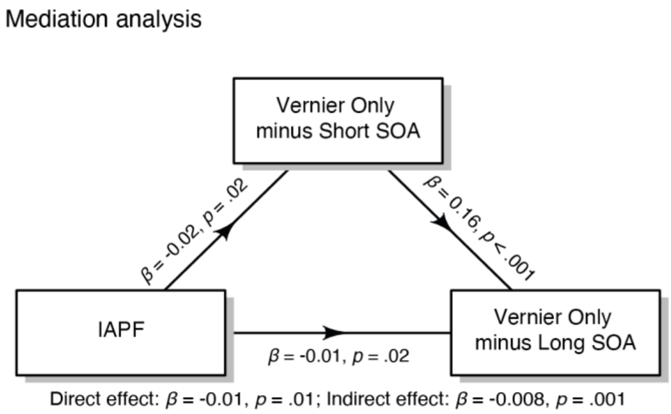

Supplementary Figure 5. Mediation analysis controlling for confounding effects due to the correlation between performance in Short and Long SOA conditions. Masking effects in the Short SOA condition (difference in performance between the Vernier Only and the Short SOA conditions) do not fully mediate the relationship between IAPF and masking effects in Long SOA (difference in performance between the Vernier Only and the Long SOA conditions; Direct effect: $\beta = -0.01$, $p = .01$).

References

Benwell, C.S.Y., London, R.E., Tagliabue, C.F., Veniero, D., Gross, J., Keitel, C., Thut, G., 2019. Frequency and power of human alpha oscillations drift systematically with time-on-task. Neuroimage 192, 101–114. https://doi.org/10.1016/j. neuroimage.2019.02.067.

Brüers, S., & VanRullen, R. (2018). Alpha Power Modulates Perception Independently of Endogenous Factors. *Frontiers in Neuroscience*, *12*, 279. https://doi.org/10.3389/fnins.2018.00279

Cohen, M. X. (2014). Fluctuations in Oscillation Frequency Control Spike Timing and Coordinate Neural Networks. *Journal of Neuroscience*, *34*(27), 8988–8998. https://doi.org/10.1523/JNEUROSCI.0261-14.2014

Menétrey, M. Q., Herzog, M. H., & Pascucci, D. (2023). Pre-stimulus alpha activity modulates long-lasting unconscious feature integration. *NeuroImage*, *278*, 120298. https://doi.org/10.1016/j.neuroimage.2023.120298

Samaha, J., & Postle, B. R. (2015). The Speed of Alpha-Band Oscillations Predicts the Temporal Resolution of Visual Perception. *Current Biology*, *25*(22), 2985–2990. https://doi.org/10.1016/j.cub.2015.10.007

Steen, J., Loeys, T., Moerkerke, B., Vansteelandt, S., 2017. medflex: an R package for flexible mediation analysis using natural effect models. J. Stat. Softw. (11), 76. https://doi.org/10.18637/jss.v076.i11.

Wutz, A., Melcher, D., & Samaha, J. (2018). Frequency modulation of neural oscillations according to visual task demands. *Proceedings of the National Academy of Sciences*, *115*(6), 1346–1351. https://doi.org/10.1073/pnas.1713318115
